# Redesigning Journal Clubs to Staying Current with the Literature

**DOI:** 10.3390/pharmacy5040062

**Published:** 2017-11-06

**Authors:** Roland N. Dickerson, G. Christopher Wood, Joseph M. Swanson, Rex O. Brown

**Affiliations:** Department of Clinical Pharmacy and Translational Science, University of Tennessee College of Pharmacy, 881 Madison Ave., Memphis, TN 38163, USA; cwood@uthsc.edu (G.C.W.); jswanson@uthsc.edu (J.M.S.); rbrown@uthsc.edu (R.O.B.)

**Keywords:** education, pharmacy practice, learning, training, journal club, critical care, parenteral nutrition, enteral nutrition

## Abstract

Staying current with the literature is of paramount importance to the pharmacist engaged in an evidence-based clinical practice. Given the expanding roles and responsibilities of today’s pharmacists combined with exponential growth in new medical and health sciences literature, staying current has become an extremely daunting task. Traditional journal clubs have focused upon their role as a training vehicle for teaching critical reading skills to residents. However, schools of pharmacy are now required to provide instruction in biostatistics, research design, and interpretation. We present a paradigm shift in the traditional journal club model whereby a collection of periodicals is screened and a short synopsis of the pertinent articles is provided. The associated tasks for screening and presenting of the primary literature are shared among a group of clinicians and trainees with similar practice interests resulting in a more reasonable workload for the individual. This journal club method was effective in identifying a significant majority of articles judged to be pertinent by independent groups of clinicians in the same practice arenas. Details regarding the shared core practice and knowledge base elements, journal club format, identification of journals, and evaluation of the success of the journal club technique are provided.

## 1. Introduction

Journal clubs are commonplace in academic medical centers and hospitals in various fields of clinical practice including pharmacy. The first record of a medical journal club was attributed to Sir William Osler at McGill University in 1875 [1]. His intent for initiation of journal club was described as “for the purchase and distribution of periodicals to which he could not afford to individually purchase” [2]. The purpose of a journal club has traditionally been described as a vehicle to teach trainees critical reading skills [2,3,4,5,6,7,8], including analysis of study design, statistical inference, and evaluation of the author’s interpretation of their findings. It also provides a forum for trainees to present the literature that they have read and interpreted. According to the Accreditation Council for Pharmacy Education’s 2016 Accreditation Standards and Key Elements for the Professional Program in Pharmacy leading to the Doctor of Pharmacy degree, coursework that includes biostatistics, ethics, research design, and health information retrieval and evaluation are required elements of the Doctor of Pharmacy curriculum [9]. Thus, the historical intent of journal clubs (e.g., to develop critical reading skills in trainees) could be less emphasized and even argued that this purpose may be antiquated for the current pharmacy residency educational environment.

Staying current with the literature is an essential requirement for the pharmacist engaged in an evidence-based clinical practice. It has been suggested that the gap between implementation of new knowledge into clinical practice is a primary reason for the suboptimal provision of quality health care for some institutions [10]. However, due to expanding responsibilities among today’s clinical pharmacy faculty and practitioners, combined with an exponential growth rate in the number of medical and health science publications [11], it becomes a daunting, if not overwhelming, task for one individual to consistently screen the abundant amount of journals to seek out those clinical studies, position papers, or clinical guidelines that may be incorporated into their evidence-based clinical practice [12]. Because of these expanding responsibilities, we have altered our journal club format from a detailed discussion of one or two articles to a methodology that allows bi-monthly screening of numerous journals pertinent to our clinical practice. We have embraced this altered format for at least the past several years within our journal club group based on the ideology of making a concerted effort to keep up to date with the literature. In addition, this format provides the opportunity for the mentoring of pharmacy residents via dialogic discussion of emerging innovative therapies. This paper reviews our current redesigned journal club format as well as the logistics to be considered in its implementation, and provides insight regarding its success in terms of staying current with the literature.

## 2. Shared Core Practice and Knowledge Base Elements of the Journal Club Participants

Five clinical faculty members with direct patient care responsibilities and three second post-graduate year (PGY2) pharmacy residents comprise the core of the journal club group. All faculty are within the same department at the University of Tennessee College of Pharmacy and are based at the same university-affiliated hospital (Regional One Health, formerly known as “The MED”, in Memphis). Advanced clinical pharmacist activities are required via a contractual agreement between the College of Pharmacy and the hospital. Two faculty members have clinical responsibilities and coordinator duties for the Nutrition Support Service (NSS), another two faculty members provide critical care pharmacy services, and one faculty member shares responsibilities for both services. The NSS faculty members provide clinical services to adult patients throughout the entire hospital that require parenteral nutrition and to select complicated patients who require enteral nutrition. The critical care faculty members provide pharmacotherapy services to a Level 1 Trauma Intensive Care Unit (TICU) and its associated step-down unit. Most of the patients managed by the NSS are also trauma and surgical intensive care unit patients that frequently coincide with the population followed by the TICU faculty. The PGY2 pharmacy residents are required to complete four months of NSS experience and four months of TICU clinical pharmacy services with the remaining months dedicated to elective experiences, research, and miscellaneous activities. Thus, all clinical faculty and PGY2 trainees share a common thread of required knowledge and reading sources necessary for optimal performance of their respective duties. It would be intuitive that both NSS and TICU clinical pharmacy services could streamline their ability to stay current with the literature by sharing reading sources and communicating findings from pertinent articles with each other.

## 3. Specific Objectives for the Journal Club

The primary intent of our journal club is to stay current with new literature that may confirm, alter, or augment conventional practice in pharmacy nutrition support or pharmacy trauma intensive care. A second objective is to accomplish the task of screening and compiling pertinent studies on a consistent basis without burdening a sole person with the excessive workload. Third, this journal club methodology provides a forum for training pharmacy resident mentees via dialogic teaching of emerging innovative therapies, where perspective on the paper’s application to clinical practice and what is currently known in the field can be provided. This model exemplifies one method on how to facilitate continued growth in knowledge of current literature for trainees to potentially emulate upon completion of their training.

## 4. Journal Club Participants and Format

The journal club process and organization are overseen by five clinical pharmacy faculty mentors. In addition to the faculty mentors, other active participants are also expected to screen journals, prepare materials for the journal club, and present papers at each session. These other participants include our three PGY2 critical care pharmacy residents and PGY1 pharmacy residents (or PGY2 pharmacy residents from another medical center hospital) enrolled in an elective NSS or TICU rotation. Students on Advanced Pharmacy Practice Experience (APPE) rotations for the month with the clinical faculty are also required to attend as non-presenting participants. Most journal club sessions are comprised of 12–15 attendees.

Journal club meetings are scheduled twice monthly on the second and fourth Tuesday of every month at 3 P.M. and is to last no longer than 1 h. This time was found to be conducive for attendance as the NSS and TICU pharmacy services tend to complete most of their clinical activities for the day by that time as clinical service responsibilities usually begin at 6 or 7 a.m. for each service. In addition, a consistent time and day facilitates the development of a routine, whereby the session is unlikely to be inadvertently forgotten and missed. The location of journal club meetings is also kept consistent and held in a conference room at the College of Pharmacy building, which is located adjacent to the hospital. The tables and chairs are arranged in a rectangular format to facilitate interactive communication among the participants. The presenter may be located within any location in the rectangle.

It has been suggested that journal clubs can encourage residents to regularly and critically read the medical literature but will not succeed if the journal club suffers from poor attendance or periodic abandonment [6]. Attendance is mandatory for the PGY2 pharmacy residents, PGY1 pharmacy residents in an elective TICU or NSS rotation, and APPE students. Attendance is not mandatory for faculty but strongly encouraged. Because faculty recognize the importance of journal clubs in their professional development as well as for the resident’s growth, they consistently attend.

Twenty-nine journals are screened monthly (Table 1). The journals are then divided into four groups with approximately the same workload in terms of collective monthly publication rate for their respective journals. The “nutrition focused” journals are compiled into a single group and presentation of that material is assigned to a pharmacy resident on NSS that month or an NSS-oriented faculty member. The other three periodical groups comprise critical care, pharmacy, infectious disease, and medical journals and is assigned to a critical care faculty member or resident on a critical care rotation. Two participants are required to present at each journal club meeting with each having up to 30 min for their presentation. The presentation schedule is arranged biannually by the PGY2 residents and the number of presentations are equally distributed among all core journal club participants. This arrangement amounts to four or five required presentations by each full-time participant yearly.

The scheduled presenter must screen the assigned journals and develop a packet of materials consisting of a face sheet, the table of contents for all assigned periodicals for the month, the first page of selected articles containing the abstract of the paper, and sometimes an entire article if the presenter considers the paper of pivotal importance. A truncated example of the journal club packet is provided in Appendix A. The face sheet contains a table containing the journal, its publication month/date, and titles of key papers selected by the presenter from those journals. The presenter is also expected to have read those key papers listed in the face sheet and be able to answer any inquiries the journal club participants may have. The current table of contents from each journal issue being presented are also provided to allow closer screening by all participants in the event a “pertinent article” was missed by the presenter. This scenario is more common among the trainee presenters as they do not have the depth of knowledge of the mentors and may not recognize an article as novel or controversial based on current information in the field. Because of the volume of material to be reviewed in thirty minutes, only a brief synopsis of those articles identified on the face sheet as “applicable to our clinical practice” can be given. The first page of the “key articles” is added to the packet of tables of contents so that the participant can read the abstract to ascertain if the full paper needs to be retrieved for closer evaluation. Additional time may be given for presentation of a paper deemed extremely important by the presenter. Under such conditions, the entire paper is usually added to the packet of materials. Only the primary literature is discussed. The preceptors will, at times, elaborate on a discussed manuscript. They will put the research in perspective based on their knowledge of the topic. This technique enhances the learning of those that are not experts in this area. For example, one preceptor might elaborate on the significance of a bacteremia study, since they personally conduct research in this area. They usually point out significant findings or deficiencies in the manuscript, based on their expertise in the field.

Reviews, guidelines, and position papers are not reviewed but may be provided on the face sheet and mentioned during the presentation to alert journal club attendees of its presence. The following month’s presenters use the current month’s face sheet to ascertain what issues of the assigned journals have already been evaluated so that a lapse in coverage in evaluating journal content can be avoided.

## 5. Identification of Journals

Our journal club’s current periodicals identified for screening and presentation are listed in Table 1. Because our practices are intertwined between NSS and TICU with the trauma intensive care unit as the primary practice focus, most selected journals are applicable to both practice settings. Since a significant contribution of the TICU pharmacy practice entails antibiotic therapy, key infectious diseases journals are additionally included. The list of periodicals intentionally includes some journals that focus on providing state of the art reviews in various critical care and nutrition support arenas. The intent of including these journals is to facilitate a broader knowledgebase in the NSS and critical care practice arenas among the journal club attendees despite the unlikelihood of presentation of specific articles. It is also anticipated that journal club attendees may independently screen and read journals not listed in Table 1. Examples of this scenario would include journals associated with their organizational memberships or periodicals that are too specialized to meet the needs of all journal club participants, or those that do not consistently provide papers applicable to our practice group but are of scholarly interest to the individual. Approximately yearly, near the end of the residency cycle, the faculty mentors convene after a journal club session to discuss which journals lacked success in meeting our educational needs and whether any journals need to be added or deleted to our current screening list of periodicals.

## 6. Evaluation of Success of the Journal Club Technique

To evaluate the success of our journal club in identifying articles pertinent to our practice, we examined a series of publications which was intended to identify the most significant articles for pharmacy nutrition support and critical care pharmacy practice published from 2013 to 2017 [12,13,14,15,16,17,18] and compared them to our current list of periodicals screened by our journal club. The intent of these publication series was to assist the pharmacist engaged in nutrition support or critical care pharmacotherapy in staying current with the pertinent literature. For identification of papers deemed important to pharmacy nutrition support practice, eight board-certified pharmacists from differing institutions with advanced practice roles and direct patient care responsibilities contributed to the collective identification of papers. The authors had different practices with some having a diverse patient population, whereas others had a focused patient population (e.g., critical care, pediatrics, oncology, and long-term/home nutrition therapy). After an initial identification of over 100 articles published per year that may be important to pharmacy nutrition support practice, the group of authors significantly reduced the number of papers deemed to be of high importance as evidenced by the majority vote (defined as at five out of eight authors agreed that the paper was of high significance) [12,13,14]. On behalf of the Critical Care Pharmacotherapy Literature Update Group, comprised of over forty critical care pharmacists across the United States, the authors reviewed hundreds of articles annually with summaries disseminated nationally via a monthly newsletter and social media outlets. Articles selected for inclusion into the yearly paper indicating major publications were rated according to the Grading of Recommendation, Assessment, Development and Evaluation (GRADE) methodology [19] in addition to consideration of their applicability to reinforce or change current clinical practice in various critically ill subpopulations. These articles were published in a series of annual publications covering the years 2012–2015 [15,16,17,18].

Eighty-seven papers significant to pharmacy nutrition support practice were identified by the author group over the four-year observation period. Seventy-five articles were from periodicals included in our journal club. An additional three articles were from a journal that was not included in the screening list of periodicals but all faculty and PGY2 residents receive as part of their membership of the journal’s sponsoring organization (Table 2). Seventy-eight out of eighty-seven papers or approximately 90% of significant papers deemed pertinent to pharmacy nutrition support practice could be potentially captured with our journal club methodology. For significant critical care pharmacotherapy papers, forty-four articles from thirteen different journals were reviewed by the Critical Care Pharmacy Literature Update Group (Table 3). Our journal club captured seven out of thirteen periodicals listed by the Critical Care group. Out of the total of forty-four identified papers from these thirteen journals that were identified by Critical Care Pharmacy Literature Update Group, our screening methodology captured 35 or 80% of them. Of the nine missed papers, seven were from cardiology journals. It is important to note that our specialized practice includes very little cardiology. Most patients with primary cardiac disorders are treated at another local hospital. The remaining journals that were not screened would be considered as low-yield journals for us throughout the year. These results indicate that a broad and appropriate selection of periodicals is of paramount importance to staying informed. Our methodology was an effective and efficient means to stay abreast of the majority of new knowledge applicable to our clinical practice.

## 7. Conclusions

Staying current with the literature is a daunting but essential task for the pharmacist who is engaged in an evidenced-based clinical practice. Presented in this review is a method for redesigning journal clubs from the traditional technique of an in-depth analysis of one or two articles to a screening tool that effectively keeps pharmacists informed of current literature. This technique reduces workload of the individual by sharing duties among multiple pharmacists and provides a forum for faculty and senior clinicians to mentor their trainees. This methodological redesign of the traditional journal club is offered to others for consideration.

## Figures and Tables

**Table 1:** Organization of journals by presentation group *.

Group	Journals
A	Antimicrobial Agents & Chemotherapy, Chest, Critical Care Clinics, Emergency Medicine Clinics of North America, Journal of Trauma, Infectious Disease Clinics of North America, Medical Clinics of North America, New England Journal of Medicine, Surgical Clinics of North America
B	American Journal of Clinical Nutrition, Annals of Surgery, Clinical Nutrition, Current Opinion in Clinical Nutrition & Metabolic Care, JPEN Journal of Parenteral and Enteral Nutrition, Nutrition, Nutrition in Clinical Practice, Surgery
C	Annals of Pharmacotherapy, Critical Care, Critical Care Medicine, Current Opinion in Critical Care, Journal of the American Medical Association, Journal of Antimicrobial Chemotherapy
D	Annals of Internal Medicine, Clinical Infectious Diseases, Intensive Care Medicine, JAMA Internal Medicine, Journal of Critical Care, Pharmacotherapy

* Groups A and B are presented on the second Tuesday of the month; Groups C and D are presented on the fourth Tuesday of the month.

**Table 2:** Evaluation of success of the journal club for identifying the most pertinent papers for pharmacy nutrition support practice from 2013 to 2016 [12,13,14].

Journal	Number of Significant Articles	Journal Screened?
Journal of Parenteral and Enteral Nutrition	27	Yes
Nutrition in Clinical Practice	10	Yes
Critical Care	7	Yes
Critical Care Medicine	7	Yes
Clinical Nutrition	7	Yes
Journal of the American Medical Association	5	Yes
New England Journal of Medicine	5	Yes
American Journal of Health-System Pharmacy	3	No *
American Journal of Clinical Nutrition	2	Yes
American Journal of Gastroenterology	2	No
Intensive Care Medicine	2	Yes
Annals of Intensive Care	1	No
Annals of Oncology	1	No
Annals of Surgery	1	Yes
British Medical Journal	1	No
Clinical Infectious Diseases	1	Yes
Diabetes Care	1	No
Journal of Pediatrics	1	No
Journal of Pediatric Surgery	1	No
Lancet	1	No
Nutrition	1	Yes
Pharmacotherapy	1	Yes

* All faculty members receive this journal as part of membership of the organization despite its not being listed in the journal club’s screening list of journals.

**Table 3:** Evaluation of success of the journal club for identifying the most pertinent papers for pharmacy critical care practice from 2012 to 2015 [15,16,17,18].

Journal	Number of Significant Articles	Journal Screened?
New England Journal of Medicine	18	Yes
Critical Care Medicine	6	Yes
Journal of the American Medical Association	5	Yes
Journal of the American College of Cardiology	4	No
Circulation	2	No
Critical Care	2	Yes
American Journal of Health-System Pharmacy	1	No *
American Journal of Respiratory and Critical Care Medicine	1	Yes
Anesthesia and Analgesia	1	No
Clinical Infectious Diseases	1	Yes
Journal of Parenteral and Enteral Nutrition	1	Yes
Lancet	1	No
Stroke	1	No

* Guideline co-published in Critical Care Medicine. All faculty members receive this journal as part of membership of the organization despite its not being listed in the journal club’s screening list of journals.

## Appendix A. Journal Club Packet Example

1. The face or cover page would contain the following information for one group of journals in this format:
  Journal Club
  Group B Journals
  9 May 2017 (*the date of the journal club presentation*)
  Roland Dickerson, Pharm.D. (*the presenter*)
  **Table A1:** Example of face page format and content.
  Journal	Month	Selected Papers of Interest
  JPEN	May	Management of parenteral nutrition (tutorial) Volume based enteral nutrition may improve caloric delivery but not clinical outcomes in critically ill patients Hyperglycemia without DM during home PN Prevention of CRBSI with catheter locks—home PN
  Am J Clin Nutr	May	None
  Clin Nutr	April	Previously done—next issue will be published in June
  Nutrition	May	Impact of nutrition support on clinical outcomes and cost effectiveness: prospective cohort with propensity score matching Age dependent risk factors for malnutrition in trauma patients Presence of validated malnutrition screening tool associated with better nutrition care
  Nutr Clin Pract	April	Proceedings of the 2016 International Protein Summit (Supplement)
  Curr Opin Clin Nutr Metab Care	May	Focus on muscle protein dynamics and pediatric nutrition Vitamin D and muscle trophicity (review)
  Ann Surg	May	Pitfalls and bias in observational studies with propensity score analysis
  Surgery	May	None
2. The next set of pages are photocopies of all assigned journals’ table of contents within the grouping for the month. The intent for providing the table of contents for all journals is for a second screening by the journal club participants to ensure all pertinent articles were identified (including those inadvertently omitted by the presenter).
3. The final set of pages are photocopies of the first page of the identified papers for each journal as outlined by the bullet points from the selected papers column on the face page. This gives the non-presenting journal club participant the ability to read the abstract to ascertain if retrieval of the full paper for more in-depth reading is necessary. Sometimes, the journal club presenter may provide the entire paper in the packet if it is deemed of high importance to our practice.
